# Seasonal patterns of gastrointestinal nematode infection in goats on two Lithuanian farms

**DOI:** 10.1186/s13028-015-0105-3

**Published:** 2015-03-19

**Authors:** Inga Stadalienė, Johan Höglund, Saulius Petkevičius

**Affiliations:** Department of Infectious Diseases, Veterinary Academy, Lithuanian University of Health Sciences, Tilžės 18, LT-47181 Kaunas, Lithuania; Department of Biomedical Sciences and Veterinary Public Health, Swedish University of Agricultural Sciences, P.O. Box 7028, , SE-750 07 Uppsala, Sweden

**Keywords:** Goats, Epidemiology, Arrested larvae, *Teladorsagia*, *Haemonchus contortus*

## Abstract

**Background:**

This study investigated seasonal changes in naturally acquired gastrointestinal nematode (GIN) infections on two Lithuanian goat farms with different parasite control practices.

**Findings:**

On both farms, nematode faecal egg counts (FEC) and larval cultures were obtained from 15 adult and 10 young goats at bi-weekly intervals from April 2012 to April 2013. Goats on farm A were dewormed with ivermectin (0.3 mg/kg body weight) in October/November 2012, whereas the animals on farm B were left untreated. Thirteen young goats were slaughtered in August/November 2012 and April 2013 and worm burdens in the gastrointestinal tract were enumerated. In goats from both farms, *Teladorsagia*, *Trichostrongylus*, *Oesophagostomum*, *Chabertia* and *Haemonchus* were the dominant GIN genera. Herbage contamination with infective third-stage larvae (L_3_) peaked in July/August and resulted in high FEC in September/October. Parasitological examination at slaughter showed that *Teladorsagia* spp. and *Haemonchus contortus* survived the winter, both in the abomasal mucosa as adults and as early fourth-stage larvae (EL_4_). Deworming on farm A significantly reduced FEC, especially of *H. contortus*, at the start of the grazing period compared with the untreated farm B (*P* < 0.05).

**Conclusions:**

Goats were heavily infected with several GIN throughout the year. Strategic anthelmintic treatment during housing significantly reduced nematode egg output, in particular by *H. contortus*, at the start of the grazing season.

## Findings

The risk of gastrointestinal nematode (GIN) infections in small ruminants is determined by factors such as climate, level of nutrition, stocking density and management. During the past two years, the goat population in Lithuania has increased by 28%, to 9,300 animals in 2014 [1]. This has led to increased stocking rates on pasture, with associated productivity losses from GIN infections [2].

The effects of GIN are predicted to become more severe due to global warming [3,4]. One such example of the possible influence of ongoing climate change is the increased infection levels of *Haemonchus contortus* observed in Scandinavia [5,6]. However, as long as anthelmintics are effective, strategic treatments reduce parasite pasture contamination and lower the exposure to GIN associated with increased milk yields in goats [7]. Deworming during housing is the most commonly used control practice against GIN in Norway [8] and it has been shown that it significantly reduces faecal egg counts (FEC) in the next grazing season [9]. Reduced FEC at the start of the grazing period prevents pasture contamination, especially by *H. contortus* since its free-living stages are sensitive to sub-zero temperatures [3,10]. *H. contortus* mainly survives the winter inside the host, as arrested forms in the abomasal mucosa [11]. Previous studies on GIN in Lithuanian sheep and cattle have shown high levels of larval inhibition [12,13]. In contrast, information from Lithuanian goats has hitherto been lacking.

This study investigated seasonal fluctuations in GIN on two goat farms in central Lithuania, of which farm A was treated with anthelmintics and farm B was left untreated. Both farms had White Shorthaired Goats. The study took place between April 2012 and April 2013 and the grazing period was from late April until late October. During housing, the animals were fed hay/haylage, vegetables (sugar-beet, carrots) and grain. The kidding period on both farms started in late January and lasted until late February. Kids were weaned after approximately 3 months. On farm A, the kids grazed separately from the adult goats until October in a paddock with 500 kg/ha (adults 320 kg/ha), whereas on farm B they grazed in the same paddock (235 kg/ha).

Mean monthly temperature was similar and peaked (19.6°C) in July. Compared with the long-term average (1961–1990), the temperature on both farms was on an average 1–3°C higher during grazing. Winter temperature varied on average between −1.0°C and −6.7°C. The highest level of rainfall was observed in June/July (89.7 − 138.6 mm).

Anthelmintics had not been used on either farm for 4 months prior to the start of the trial. On farm A, the young goats received injectable ivermectin (Ivomec® 1%, 0.3 mg per kg body weight) in early October, while the adults were dewormed in late November. On farm B, all goats were left untreated throughout the study.

The work was performed in compliance with Lithuanian animal welfare regulations (No. B1-866, 2012; No. XI-2271, 2012) and was approved by the Lithuanian Committee of Veterinary Medicine and Zootechnics Sciences (Protocol No.07/2010).

Animals in each flock were selected by stratified random sampling by sex (female) and body weight, and categorised as young goats (<1 year; n = 10) or adults (>1 year; n = 15). Faeces samples were collected directly from the rectum at bi-weekly intervals. FEC were performed using a modified McMaster technique with minimum detection level of 20 nematode eggs per gram (EPG) faeces [14]. Furthermore, 1 g samples of faeces from each animal in the same grazing group were pooled and faecal cultures were prepared to obtain infective third-stage larvae (L_3_) [15]. Identification to genus or species level was based on morphological keys [16]. In addition, triplicate (≈400 g) samples of herbage were collected between May and November 2013 from each of the paddocks used by the goats on both farms, for determination of number of L_3_ [17]. Furthermore, six young females from farm A and seven from farm B were sent to the local slaughter-house for slaughtering between 25 August and 25 April 2013 and the viscera of the goats were collected for parasitological investigation. The abomasa and small- and large intestines were opened for enumeration and identification of GIN, while the abomasal mucosa was digested and examined for inhibited stages according to Grønvold [18]. Nematodes were collected and identified to species or genus [19]. Statistical comparisons of FEC between farms were performed using Repeated Measures Analysis of Variance (ANOVA) analysis in BMI SPSS Statistics 21 version. Prevalence of nematode infections in the gastrointestinal (GI) tract and standard 95% confidence intervals (CI) were also calculated. Worm burdens on the two farms were compared using one-way ANOVA, with *P* < 0.05 as statistically significant.

**Figure 1:**
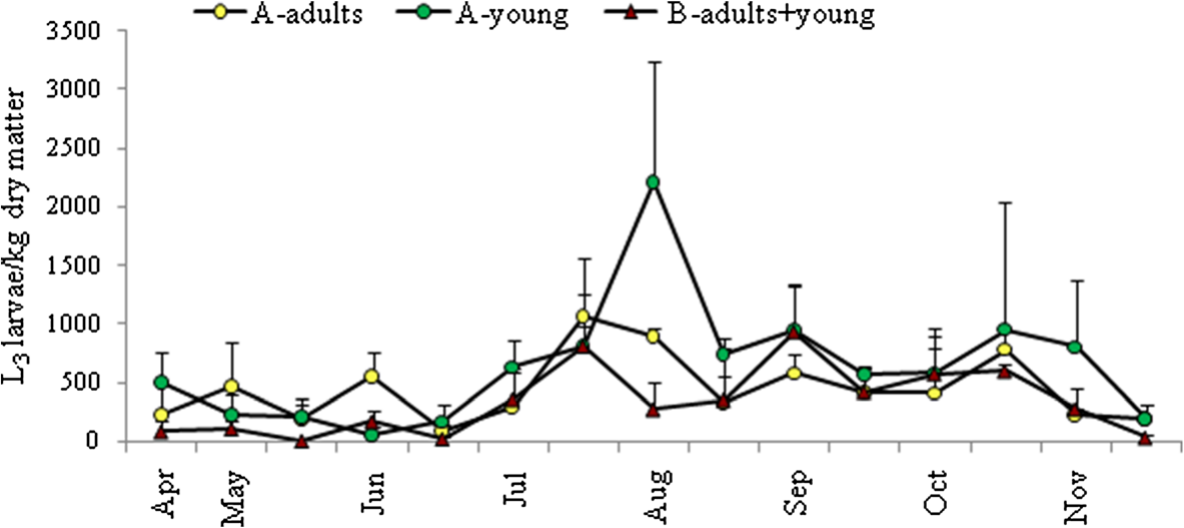
**Mean number of L**_**3**_ **stage larvae per kg dry matter of grass on farms A and B.**

**Figure 2:**
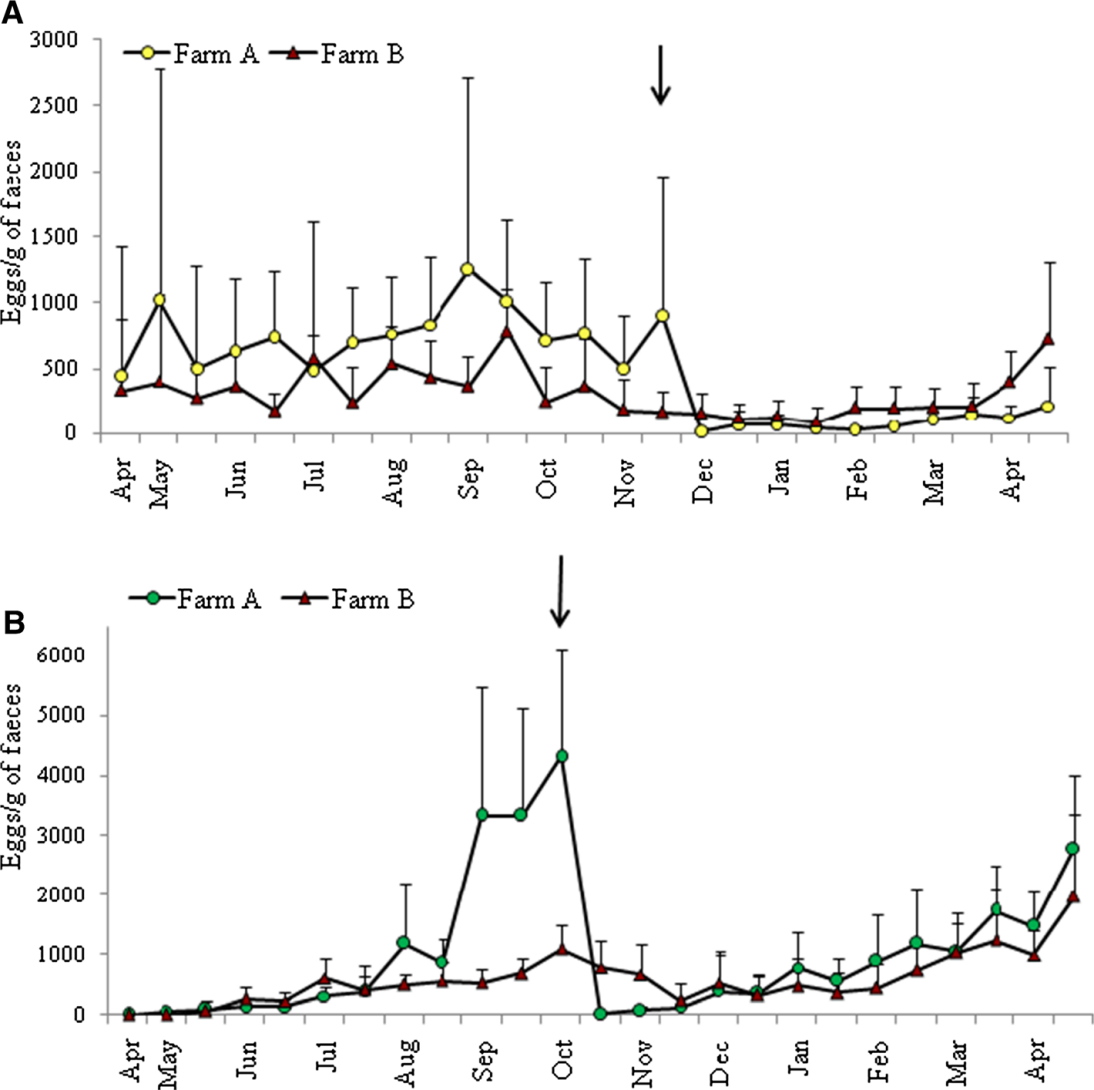
**Mean number of strongyle nematode eggs per gram faeces (EPG) in A) adult dairy goats and B) young goats on anthelmintic-treated farm A and untreated farm B.** Arrows indicate time of treatment on farm **A**.

Pasture contamination was low (88–499 L_3_/kg) at the start of the study and remained low until late June (Figure 1). In contrast, from late June, L_3_ numbers in herbage began to increase, with a peak in late July, in the paddock used by adult goats on farm A and in the common paddock on farm B. Two weeks later, in August, there was a peak in L_3_ pasture contamination in the paddock grazed by the young goats on farm A. These peaks in L_3_ between July and August represented the first wave of pasture contamination, and must have originated from nematode eggs shed with the faeces of adult goats between April and June (Figure 2A). These parasite eggs most likely originated from adult worms that had resumed their development from arrested larvae in the mucosa of adult goats. This infection wave resulted in subsequent peaks of parasite eggs shed, which were observed in both adult flocks in September. However, the FEC was much higher on farm A, probably as a result of the high stocking density. The FEC in young goats on both farms peaked and reached its highest levels in September/October 2012 (Figure 2B). However, again FEC was significantly higher (*P* < 0.001) on farm A, probably as a result of the higher grazing intensity compared with farm B. All young goats on farm A were dewormed in October 2012. Although this decreased FEC, it did not prevent the spring rise in FEC, which was observed towards the end of April in the next year. The second wave of pasture contamination in October resulted in re-infection and a source of arrested larvae, creating an overwintering nematode population in the GI tract. During the housing period (November − April), FEC in the young goats gradually increased, followed by a marked rise by the end of April 2013 on both farms.

*Teladorsagia* dominated both in the samples from pasture (42 − 100%) and in faecal cultures (42%) on both farms, confirming results from other European countries [6,20-22]. In addition, L_3_ of *Trichostrongylus* (26%), *Oesophagostomum* (13%), *Chabertia* (11%) and *H. contortus* (8%) were identified in the faecal cultures. Interestingly, *H. contortus* was only observed during the grazing period (May-October) on farm A (3 − 35% of the total nematode population) and farm B (8 − 31%). In addition, *H. contortus* was detected in faecal cultures of young goats from late June and increased between August and October. This was particularly the case on farm A, where *H. contortus* comprised 16 − 21% of the total nematode population. Similarly, pasture contamination with *H. contortus* larvae was first detected in mid-May, but was highest between August and October (9 − 50%). This is in agreement with Swedish findings on GIN in sheep [10].

In the GI tract, the following species were identified in the abomasum: *Teladorsagia circumcincta* 52% (95% CI: 42 − 62%), *Te. trifurcata* 34% (95% CI: 23 − 47%), *Trichostrongylus axei* 7% (95% CI: 3 − 12%) and *H. contortus* 7% (95% CI: 1 − 12%). In the small intestine, the species identified were: *T. capricola* 45% (95% CI: 34 − 56%), *T. colubriformis* 43% (95% CI: 32 − 52%), *T. vitrinus* 11% (95% CI: 7 − 16%) and *Strongyloides papillosus* 1% (95% CI: 0 − 2%). The species found in the large intestine were: *Oesophagostomum venulosum* 65% (95% CI: 52 − 80%), *Chabertia ovina* 34% (95% CI: 19 − 49%) and *Trichuris ovis* 1% (95% CI: 0 − 3%). For *Teladorsagia* spp., adult worms dominated in the abomasum, while for *H. contortus* early fourth-stage larvae (EL_4_) dominated (Table 1). Arrested *H. contortus* were mainly observed between August and November (53 − 99%), but to a lesser extent also in early April (Table 1). Developing larvae (DL_4_) of *H. contortus* were found until late October and of *Teladorsagia* spp. until late November. Subsequent examinations in April showed that ≥95% of the total worm burdens were adults, with only a low percentage of *H. contortus* (3%) and *Teladorsagia* (4%) in the EL_4_ stage. The L_3_ of *H. contortus* ingested in autumn obviously did not start to develop until early spring, resulting in decreased L_4_ development and adult worm reproduction. Tracer tests on lambs in Sweden have shown that arrest of *H. contortus* takes place mainly from July and that *T. circumcincta* comprises a lower percentage of the increasing numbers of nematodes during the grazing season [11]. The significantly higher numbers of adults than of developing stages (*P* < 0.05) of *Trichostrongylus* spp., *O. venulosum* and *C. ovina* observed in the tracers indicate that these nematodes survived the winter within the host as adults (Table 2). Deworming of adult goats at the end of November on farm A significantly reduced FEC at the start of grazing compared with on the untreated farm B (*P* < 0.05). After deworming on farm A, *H. contortus* L_3_ were not observed in faecal cultures until March 2013 and in April comprised only 2–3%. The corresponding proportion of this parasite in faeces of goats on farm B was 1–4% during housing, but in April of the next year 38% of the cultured L_3_ were *H. contortus*. Thus, deworming of adults and young goats in late autumn reduced the *H. contortus* population considerably in early spring and thereby prevented pasture contamination. The high percentage of *H. contortus* (31-53%) in faecal cultures of adult and young goats shows that development of this parasite resumed from April–May. If deworming is not performed in late autumn, it could be implemented in spring before turn-out [5].

**Table 1:** **Mean number of worms and proportion (% of total) of** ***Haemonchus contortus*** **and** ***Teladorsagia*** **sp. development stages in abomasum of young goats at slaughter**

	***H. contortus***				***Teladorsagia*** ^**a**^			
**Date**	**Adult**	**DL**_**4**_	**EL**_**4**_	**Mean total**	**Adult**	**DL**_**4**_	**EL**_**4**_	**Mean total**
25 Aug	134 (20%)	54 (6%)	553 (74%)	741	4949 (83%)	154 (3%)	908 (14%)	6011
14 Sep	71 (1%)	0	8325 (99%)	8396	10346 (66%)	451 (3%)	4500 (31%)	15297
08 Oct	300 (22%)	225 (25%)	251 (53%)	776	4522 (93%)	111 (2%)	183 (4%)	4816
29 Oct	0	24 (1%)	3656 (99%)	3680	5633 (86%)	74 (1%)	826 (13%)	6533
29 Nov	239 (12%)	0	2342 (88%)	2581	7196 (88%)	12 (1%)	1026 (11%)	8234
25 Apr	485 (97%)	0	14 (3%)	499	1083 (96%)	0	46 (4%)	1129

^a^ *Te. circumcincta* and *Te. trifurcata*.

**Table 2:** **Mean number of worms and proportion (% of total) of** ***Trichostrongylus*** **and** ***Oesophagostomum/Chabertia*** **development stages in the small and large intestines of young goats at slaughter**

	***Trichostrongylus*** ^**a**^			***Oesophagostomum/Chabertia*** ^***b***^		
**Date**	**Adult**	**Developing**	**Mean total**	**Adult**	**Developing**	**Mean total**
25 Aug	2851 (75%)	1067 (25%)	3919	163 (20%)	687 (80%)	850
14 Sep	15005 (91%)	1000 (9%)	16305	515 (83%)	130 (17%)	645
08 Oct	35174 (87%)	4073 (13%)	39247	78 (48%)	98 (52%)	176
29 Oct	4697 (98%)	87 (2%)	4784	235 (92%)	20 (8%)	255
29 Nov	4962 (93%)	419 (7%)	5381	105 (78%)	30 (22%)	135
25 Apr	8775 (100%)	0	8775	240 (100%)	0	240

^a^ *T. capricola, T. colubriformis* and *T. vitrinus*.

^b^ *O. venulosum* and *C. ovina*.

In conclusion, the Lithuanian goats studied here were infected with a mixture of GIN, in particular *Teladorsagia* spp. but also several other genera, including the more pathogenic *H. contortus*. FEC fluctuated in relation to the level of herbage contamination, which varied according to season on both farms. Strategic anthelmintic treatment of adult goats in November significantly reduced FEC, especially of *H. contortus.*
